# The effect of on-line position correction on the dose distribution in focal radiotherapy for bladder cancer

**DOI:** 10.1186/1748-717X-4-38

**Published:** 2009-09-24

**Authors:** Dominique C van Rooijen, Jeroen B van de Kamer, René Pool, Maarten CCM Hulshof, Caro CE Koning, Arjan Bel

**Affiliations:** 1 Department of Radiation Oncology, Academic Medical Center, Amsterdam, The Netherlands

## Abstract

**Background:**

The purpose of this study was to determine the dosimetric effect of on-line position correction for bladder tumor irradiation and to find methods to predict and handle this effect.

**Methods:**

For 25 patients with unifocal bladder cancer intensity modulated radiotherapy (IMRT) with 5 beams was planned. The requirement for each plan was that 99% of the target volume received 95% of the prescribed dose. Tumor displacements from -2.0 cm to 2.0 cm in each dimension were simulated, using 0.5 cm increments, resulting in 729 simulations per patient. We assumed that on-line correction for the tumor was applied perfectly. We determined the correlation between the change in D_99% _and the change in path length, which is defined here as the distance from the skin to the isocenter for each beam. In addition the margin needed to avoid underdosage was determined and the probability that an underdosage occurs in a real treatment was calculated.

**Results:**

Adjustments for tumor displacement with perfect on-line position correction resulted in an altered dose distribution. The altered fraction dose to the target varied from 91.9% to 100.4% of the prescribed dose. The mean D_99% _(± SD) was 95.8% ± 1.0%. There was a modest linear correlation between the difference in D_99% _and the change in path length of the beams after correction (R^2 ^= 0.590). The median probability that a systematic underdosage occurs in a real treatment was 0.23% (range: 0 - 24.5%). A margin of 2 mm reduced that probability to < 0.001% in all patients.

**Conclusion:**

On-line position correction does result in an altered target coverage, due to changes in average path length after position correction. An extra margin can be added to prevent underdosage.

## Background

External beam radiotherapy is a common treatment for muscle-invasive bladder cancer for patients unfit or unwilling to undergo a radical cystectomy. Unifocality of the tumor allows irradiation of only the tumor and to spare the healthy part of the bladder, or to give the tumor a high dose with a concomittant boost technique while giving the healthy bladder a prophylactic dose [1,2]. However, the position of a bladder tumor varies markedly day-to-day, up to several centimeters, due to bladder and bowel movements and bladder filling [3-8]. For radiotherapy, large margins are necessary to compensate for this uncertainty, resulting in a high dose to the surrounding healthy tissue. The probability of serious complications in the healthy tissue limits the dose that can be administered to the tumor. Since bladder tumors move independently of the bones, a portal image of the pelvic bones is not suitable for determining the current position of a bladder tumor [5].

Fortunately, the possibilities for image guided radiotherapy (IGRT) have increased substantially in the last decade and a megavoltage image of the bony anatomy is no longer the only option. The recent development of a cone-beam CT (CBCT) mounted on the linear accelerator enables in-room soft-tissue visualization [9]. However, the soft-tissue contrast is poor and to visualize the bladder tumor effectively markers have to be inserted around the tumor. The combination of CBCT and implanted markers opens up the way for on-line position correction of bladder tumors [10,11]. This technique enables margin reduction, leading to a decreased risk of healthy tissue complications and it might enable dose escalation.

**Figure 1:**
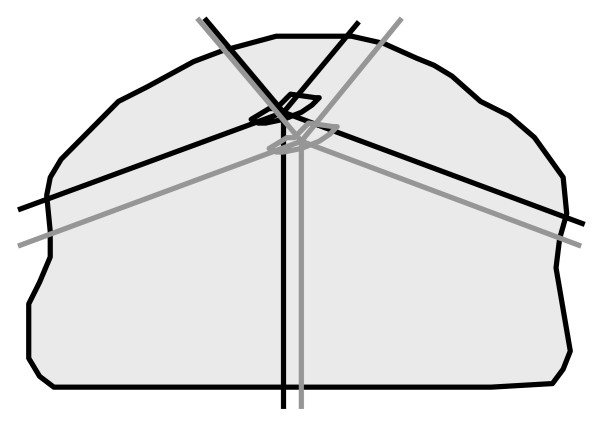
**Schematic representation of a simulation**. The black target and beam axes represent the treatment plan. Grey represents the situation on a particular treatment day. The tumor has moved within the body and position correction is applied to ensure that the beams are at the same location with respect to the target.

Applying on-line position correction causes beams to pass through a different amount of tissue and through different tissue types compared to the treatment plan, resulting in an altered attenuation for these beams (figure 1). The question arises: how is the target dose distribution affected by these differences in attenuation with respect to the treatment plan?

Similarly, an effect of on-line position correction on the target dose for the prostate has been reported [12-14]. Several authors studied the target coverage of the prostate after applying translational position correction. They concluded that on average the target dose after position correction is higher than without correction, but lower than in the treatment plan. All studies explained the remaining underdosage by rotations that were not included in the correction and by deformations. Van Herten et al. have shown that when there is no correction for rotations, the target coverage of the prostate is still 98.7% and for the seminal vesicles 95.7%, even though the planning was done without PTV margin [15]. Orton and Tomé neglected rotations of the prostate and they found that the dose distribution after applying position correction was nearly identical to the treatment plan [16].

However, the prostate is located relatively central with respect to the body contour, while bladder tumors can also be present on the ventral side of the bladder. This might have a considerable effect on the dose distribution when the tumor moves. In addition, the typical day-to-day variation in the position of a bladder tumor is larger than that of the prostate. Therefore the above mentioned results do not automatically apply for on-line position correction for bladder cancer.

In this study we investigated the effect of on-line position correction for bladder tumors on the dose distribution. The goal of this study was to determine what the effect of on-line position correction of bladder tumors is on the dose distribution, how it can be predicted and how it can be handled. In order to put the results into perspective, we added the results of the analysis of tumor movement in recently treated bladder cancer patients in our department.

## Methods

### Patients

This study included 25 patients with a histologically proven bladder tumor who were previously treated in our department. For all patients a planning CT with 3 mm slices was acquired with the patient in the supine position. The tumor was delineated by an experienced radiation oncologist. The average tumor volume was 48.8 cc (range: 7.4 - 116.0 cc). The delineated tumor volume was used as the clinical target volume (CTV) [17]. Bladder and rectum were also delineated. Because we wanted to investigate the effect of on-line position correction only and were not interested in all other uncertainties and errors, we did not use a CTV-PTV margin in this simulation study.

### Treatment planning

IMRT plans with five beams were generated for each patient: 40°, 110°, 180°, 250° and 320°. We used PLATO (Nucletron BV, Veenendaal, the Netherlands) for treatment planning. The dose calculation was based on Bortfeld's pencil beam algorithm [18] and we corrected for inhomogeneities, using an improved version of the equivalent tissue-air ratio (ETAR) correction [19]. The requirement of the plans was that 99% of the volume of the CTV was covered by the 95% isodose. This plan was only generated for study purposes. The patients were actually treated with our current technique and a CTV-PTV margin [1,17].

### Simulation

The treatment plan was made with the tumor in the original position as outlined on the planning CT. In the simulation the tumor was shifted to another position and perfect position correction was applied, i.e. the beam positions were kept constant with respect to the target. This is schematically represented in figure 1. All other parameters, for example the beam geometry and the number of monitor units, were kept constant. We simulated shifts from -2.0 to 2.0 cm in 0.5 cm increments in the left-right, cranial-caudal and dorsal-ventral direction and all combinations, yielding 9^3 ^= 729 shifts. The dose distribution was calculated for each shift. A stand-alone version of PLATO's dose engine was used for these computations. This PC version of PLATO was highly optimized for fast dose calculations on a graphical card [20].

### Analysis

The dose at least received by 99% of the volume (D_99%_) of the CTV was evaluated. If the D_99% _was lower than 95%, it was considered an underdosage. Differences in the dose distribution are caused by changes in path length and to a lesser degree by changes in tissue type. The SSD of beams might change, as can be seen in figure 1, and the beams may pass through bone which they did not in the treatment plan, or vice versa. We investigated the correlation between the dose and the average path length of the five beams, using SPSS 16.0.2. (SPSS Inc., Chicago, USA). Both the dose and the path length were calculated relative to the treatment plan in order to evaluate the results for all patients at once. We calculated the linear regression of dose versus path length for the results of all patients.

A distinction was made between the physical path length (PPL) and the radiological path length (RPL). By physical path length we mean the path length from the skin to the isocenter for the central beam axis in centimeters. The radiological path length is this length corrected for the relative electron density and it represents the equivalent path length in water.

Besides calculating the average path length of the five beams of each simulation, the physical path length weighted over the number of monitor units of each beam (PPL_MU_) was also determined:

(1)

where PPL_i _is the physical path length of beam i and MU_i _is the number of MUs of beam i and n is the number of beams. The radiological path length weighted over the number of MUs per beam (RPL_MU_) was calculated by substituting RPL for PPL in equation 1. The correlation coefficient was used to determine which of the four predictors (PPL, RPL, PPL_MU _or RPL_MU_) was the best predictor of the dose change at the target.

### Margin

We investigated what margin was needed to have avoided underdosage despite perfect position correction in the two 'worst case' patients from our data analysis, i.e. the two patients with the lowest D_99% _in a single fraction. An isotropic CTV-PTV margin of 2 mm was used. New IMRT plans were made with this margin and the full simulation was repeated for these patients. If this margin was not sufficient, the margin was increased by 1 mm increments and the planning and simulation were repeated until a satisfactory margin was obtained.

### Analysis of tumor movement and probability of underdosage

In order to calculate the probabilities of each shift to occur in an actual treatment, the movement of the bladder tumor in recently treated bladder cancer patients in our department was determined. The displacement of the tumor with respect to the bones was analyzed in patients with implanted markers from whom at least 6 CBCTs were available. These data were supplemented with the data of patients without markers, but with at least 6 repeat CTs. For the latter group the tumor was delineated in each repeat CT and the displacement of the center of gravity (CoG) of the tumor with respect to the bony anatomy was analyzed. Because the image quality of the CBCT is not sufficient for delineation, we could only use patients with enough (at least 6) regular CTs for the group without markers. We determined the systematic and random component of the tumor movement with respect to the bones. For that purpose, we first determined the individual mean and standard deviation for each patient, using translations only, with XVI release 3.5 (Elekta, Crawley, UK). The systematic component of the tumor movement in all patients is the SD of the individual means and the random component is the root mean square of the individual SDs.

Based on these tumor displacement data, the probability that a systematic underdosage occurred was calculated for each patient. First, the probability that a simulated shift could be the systematic displacement of a real treatment was calculated. Next we determined for each patient which shifts resulted in an underdosage. The probability of all shifts that led to an underdosage were summed for each patient, which gave the probability that a systematic underdosage occurred. Note that we did only simulate the effect of systematic errors, not that of additional random errors.

From the tumor displacement data the margin that would be necessary if no position correction was applied can also be determined, using Van Herk's margin recipe [21]:

(2)

where **m **is the margin, **Σ **the systematic tumor displacement and **σ **the random tumor displacement. This margin was compared to the margin that was needed when on-line position correction was applied.

## Results

### Dose

**Figure 2:**
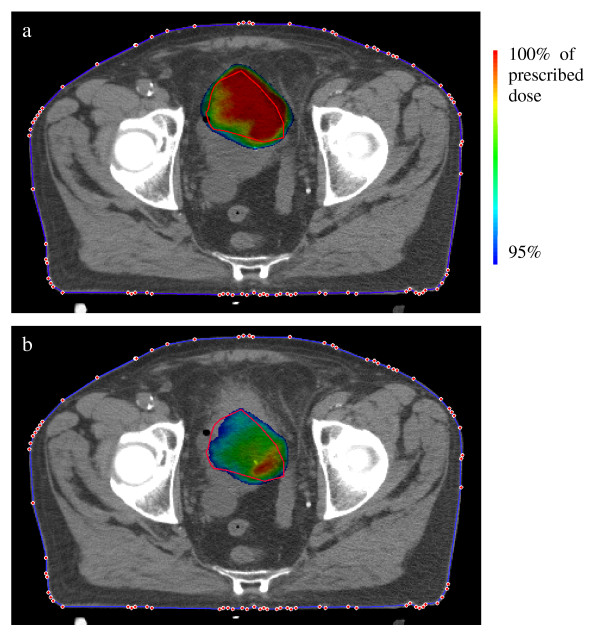
**Dose distribution at the target**. (a) Planning situation. (b) Situation when the tumor moved 2 cm dorsal and the patient position was corrected. After position correction the resulting dose is lower and shaped differently compared to the treatment plan of this scenario.

**Figure 3:**
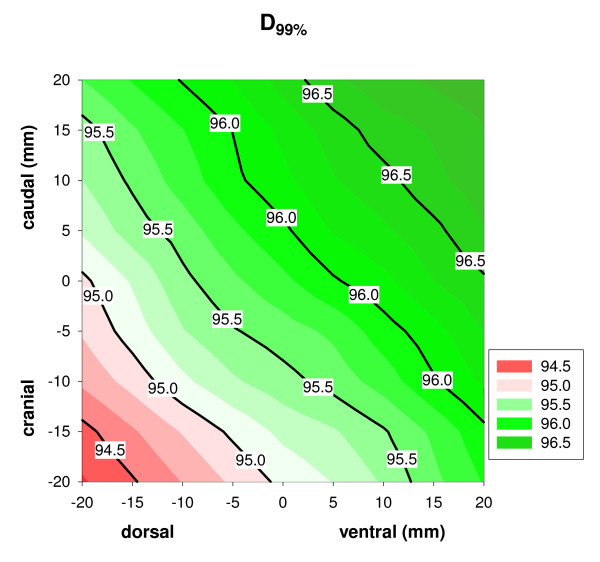
**D_99% _over the dorsal and ventral axes**. Values of D_99% _for all shifts over the dorsal-ventral and cranial-caudal axes averaged over all patients. The X-axis represents tumor displacement in the dorsal-ventral direction and the Y-axis the cranial-caudal direction. There is no shift in the left-right axis in this case. The dose increases when the tumor moves in the caudal and ventral direction. The dose decreases when the tumor moves in the cranial and dorsal direction.

When the tumor was displaced with respect to the body contour and position correction was applied perfectly, the dose distribution changed with respect to the treatment plan. These changes showed up both in the magnitude of D_99% _as well as in the shape of the 95% isodose. Note that this could also imply a dose increase. Figure 2 shows an example of the dose distribution in a treatment plan and the dose distribution when the tumor has moved 2.0 cm in the dorsal direction. In general, the dose increased when the tumor moved in the caudal and ventral directions and the dose decreased when the tumor moved in the cranial and dorsal directions (figure 3). The result of displacement in the left-right direction was patient-dependent, probably dependent on the location of the tumor on the bladder wall.

**Figure 4:**
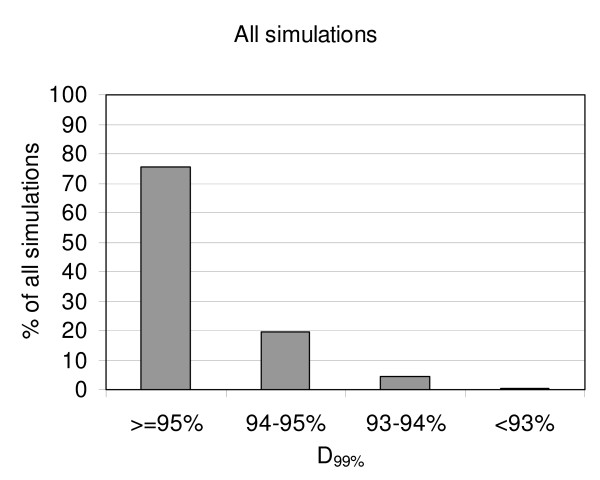
**Distribution of D_99% _for all simulations**. The X-axis represents ranges of D_99% _and the Y-axis the percentage of simulations that are in each range. Only 0.4% of all simulations had a D_99% _below 93%, with a minimum of 91.9%.

The D_99% _was at least 95% of the prescribed dose in 75.5% of all the simulated situations and at least 94% of the prescribed dose in 95.2% of the simulations (figure 4). The lowest D_99% _observed in all simulations was 91.9%, the highest was 100.4% and the mean D_99% _(± SD) was 95.8% ± 1.0%.

### Correlation between dose and path length

**Figure 5:**
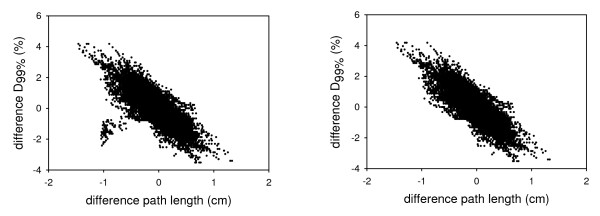
**Correlation difference D_99% _and path length change**. a: The results of all simulations of all 25 patients with respect to the treatment plan. On the X-axis is the difference in path length weighted with MUs (PPL_MU_) with respect to the treatment planning and on the Y-axis is the difference in dose with respect to the treatment plan. b: The results when patient P is excluded.

The correlation coefficient R^2 ^was 0.51 (p < 0.001), 0.25 (p < 0.001), 0.59 (p < 0.001) and 0.25 (p < 0.001) for the correlation for all patients between dose and PPL, RPL, PPL_MU _and RPL_MU_, respectively. Figure 5a shows the result of the relative dose difference and PPL_MU _for all patients. One patient, denoted patient P, had aberrant results; the slope of the regression curve was 1.22, whereas the mean slope (± SD) of the other individual regression curves was -2.39 ± 1.53. When patient P was excluded from the analysis, R^2 ^= 0.67 (p < 0.001) for the correlation between dose and PPL_MU _(fig 5b).

### Margin

Additional treatment plans with small margins were made for the two worst case patients and the simulation was repeated. The result of replanning with an increased margin was an increase of the minimum dose to the tumor, also in the treatment plan. For the worst patient a margin of 3 mm was necessary to avoid underdosage and for the other patient a margin of 2 mm was sufficient.

### Analysis of tumor movement and probability of underdosage

We determined the systematic and random components of the tumor movement with respect to the bony anatomy in each direction. For five patients the CBCTs were analyzed, with an average of 7.8 scans per patient. For 16 patients the regular CTs were analyzed, with an average of 8.6 repeat CTs per patient. Systematic displacement Σ_LR _(left-right), Σ_DV _(dorsal-ventral) and Σ_CC _(cranial-caudal) were 1.8 mm, 4.7 mm and 4.1 mm, respectively. Random displacement σ_LR_, σ_DV _and σ_CC _were 1.7 mm, 3.4 mm and 3.5 mm, respectively. If no position correction would be applied a margin of 5.7 mm, 14.2 mm and 12.7 mm in the left-right, dorsal-ventral and cranial-caudal direction, respectively, would be needed to compensate for tumor displacement, using Van Herk's margin recipe [21].

With the above SDs of systematic tumor displacement, we calculated the probability of a systematic underdosage (i.e., a D_99% _lower than 95% of the prescribed dose) if no margin was applied for each patient. When no margin was applied, the median of this probability was only 0.23%. For 16 out of the 25 patients this probability was below 1%, for 8 patients the probability was between 1% and 14% and one patient had a probability of 24.5%. The probability of a D_99% _below 94% was 0.07% in the worst case patient and smaller than 0.01% in all other cases. When a margin of 2 mm was applied, the probability of a D_99% _lower than 95% was smaller than 0.001% in the one patient for whom 2 mm was not sufficient for all simulated shifts.

## Discussion

The goal of this study was to investigate whether underdosage still occurs with perfect on-line position correction and if so, under what conditions it occurs and how it can be dealt with. We have shown that even if on-line position correction is applied perfectly, underdosage can still occur. There was a linear correlation between the dose difference and the change in averaged path length of the beams. A margin can be added to prevent this underdosage.

Although the tumor is underdosed in some situations, the lowest D_99% _that occurred in this study was still 91.9%. This represents the worst case of all 18225 simulations and was calculated for a shift of 2 cm cranially, 2 cm dorsally and 2 cm to the left. However, this result is much better than not applying position correction at all. With the systematic displacement of the tumor that we found in our patients, it is highly unlikely that this will be the systematic error of a treatment.

For every patient we calculated what the probability was that a systematic underdosage occurred in a real treatment. The range of probabilities that were found is large, 0-24.5%, but in most cases that probability is small and the median is 0.23%. The probability that the dose is systematically below 94% is 0.07% in the worst case and smaller than 0.01% in all other cases.

In this study we focused on D_99% _and every result below 95% was considered an underdosage, but for the clinical outcome the degree of underdosage is more relevant. Tomé and Fowler found in a modelling study that an underdosage in a subvolume as small as 1% of the volume of the tumor can already decrease the tumor control probability (TCP) [22]. However, this TCP decrease was apparent only if the dose in that subvolume was more than 10% lower than the prescribed dose. With a minimal D_99% _of 91.9% a decrease in TCP is not likely in our study.

Overdosage, on the other hand, might be a risk too, because constraints for organs at risk (OARs) can be violated. However, the dose increases when the tumor moves in the ventral and caudal direction, while the OARs - the rectum and small intestines - are located on the dorsal and cranial side of the bladder, respectively.

The change in dose with respect to the treatment plan can be predicted using the number of MUs per beam and the change in physical path length. Considering the radiological path length of the central axis only is apparently not the best predictor for dose differences, although this might seem counterintuitive. A likely explanation is that the central axis ray of each beam is not representative of the change in radiological path length of the entire beam. The correlation between weighted path length and dose did not hold for one of the 25 patients: patient P. A more detailed analysis of patient P showed that the tumor was highly elongated. When the dose in one part of the tumor increased as expected, the dose in another part of the tumor decreased, causing an overall decrease in D_99%_. In general the change in path length is a good predictor for the change in dose, but in practice its applicability will be limited.

It would be interesting to perform a similar study with daily CBCTs and investigate how large the dose difference over the whole treatment is with the real body contour and tumor position. Presently, not enough daily CBCTs are available in our institution to perform such an analysis. Furthermore, we cannot delineate the tumor on the CBCT, because of the poor image quality caused by scatter. The position of the tumor can be found using the markers, but in the case of deformation the shape cannot be determined. Another problem related to the scatter is that we cannot rely on the CBCT for dose calculation, because the grey values of the CBCT do not correspond to those of the CT. Moreover, the grey values are also related to the size of the patient and therefore it is hard to assign correct electron densities to the hounsfield units. Recent studies have shown that the errors in dose calculation for CBCT decrease from 10-20% to 1-2% when correction techniques are applied, which is very promising for future dose calculation for CBCT [23,24].

Without on-line position correction a margin of 5.7 mm left-right, 14.2 mm dorsal-ventral and 12.7 mm in the cranial-caudal direction would be needed to compensate for tumor displacement. This is sufficient to ensure that 90% of the patient population receives a dose of 95% or higher in the CTV. When on-line position correction is used, a margin of 2 mm would be sufficient in more than 99.99% of the patients. However, margins are not only meant for compensation of target translations, but they are also needed for uncertainties that cannot be corrected by on-line position correction for translations, such as deformations, rotations, intrafraction motion and delineation uncertainties. Because we only wanted to study the effect of on-line position correction all these errors were ignored, but they should not be forgotten in clinical practice. Engels et al. have already shown a reduced treatment outcome for patients with implanted markers and daily positioning and they also warn not to reduce margins too much [25].

The minimal D_99% _in this study was 91.9%, which is not that much lower than the required 95%. Furthermore, it is highly unlikely that the lowest D_99% _of this study will occur in a real patient. The modelling study of Tomé and Fowler suggests that our minimal D_99% _will not reduce the TCP [22]. We therefore recommend not to increase the margins to compensate for underdosage due to path length changes. Note that all other uncertainties should be adequately covered.

## Conclusion

The purpose of this study was to investigate the dosimetric effect of on-line position correction. The conclusion is that the dose distribution changes with respect to the treatment plan, even if the target is not deformed and does not rotate. However, the occurring underdosage is small, D_99% _is 91.9% in the worst case. The probability that an underdosage occurs in an actual treatment is small for most patients, but with a large spread: median 0.23%, range 0-24.5%. With a margin of 2 mm, the probability of underdosage is < 0.001% for all patients.

## Competing interests

This work was supported by a grant from Elekta.

## Authors' contributions

DR made the IMRT plans for this study, did the simulations and the statistical analysis and is the main author of the manuscript. JK gave support with treatment planning and the design of the study. RP and AB provided the software for the simulation. MH delineated the structures necessary for treatment planning. AB gave support with the statistics. AB, CK and JK were the senior researchers and provided coordination during the study. JK, RP, MH, CK ad AB reviewed the manuscript. All authors have read and approved the manuscript.
